# Supplementary material for: The open to closed D-loop conformational switch determines length in filopodia-like actin bundles
Source: Biochem J. 2024 Dec 23;481(24):1977–95. doi: 10.1042/BCJ20240367 (PMC11668490; doi:10.1042/BCJ20240367)
Supplement: Supplementary Material 1 [file BCJ-481-1977-s1.pdf]

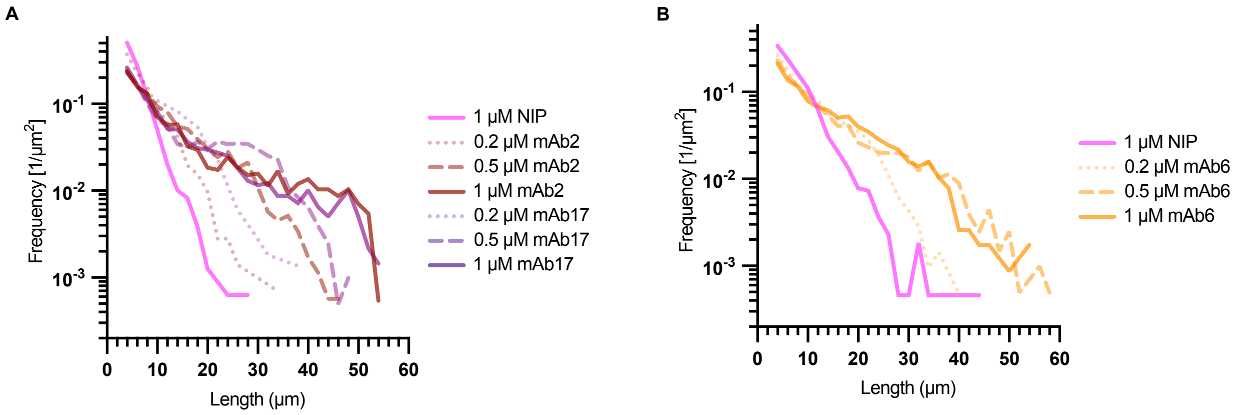
A
B
Frequency [1/μm2]
Length (μm)
1 μM NIP
0.2 μM mAb2
0.5 μM mAb2
1 μM mAb2
0.2 μM mAb17
0.5 μM mAb17
1 μM mAb17
0.2 μM mAb6
0.5 μM mAb6
1 μM mAb6

Figure S1

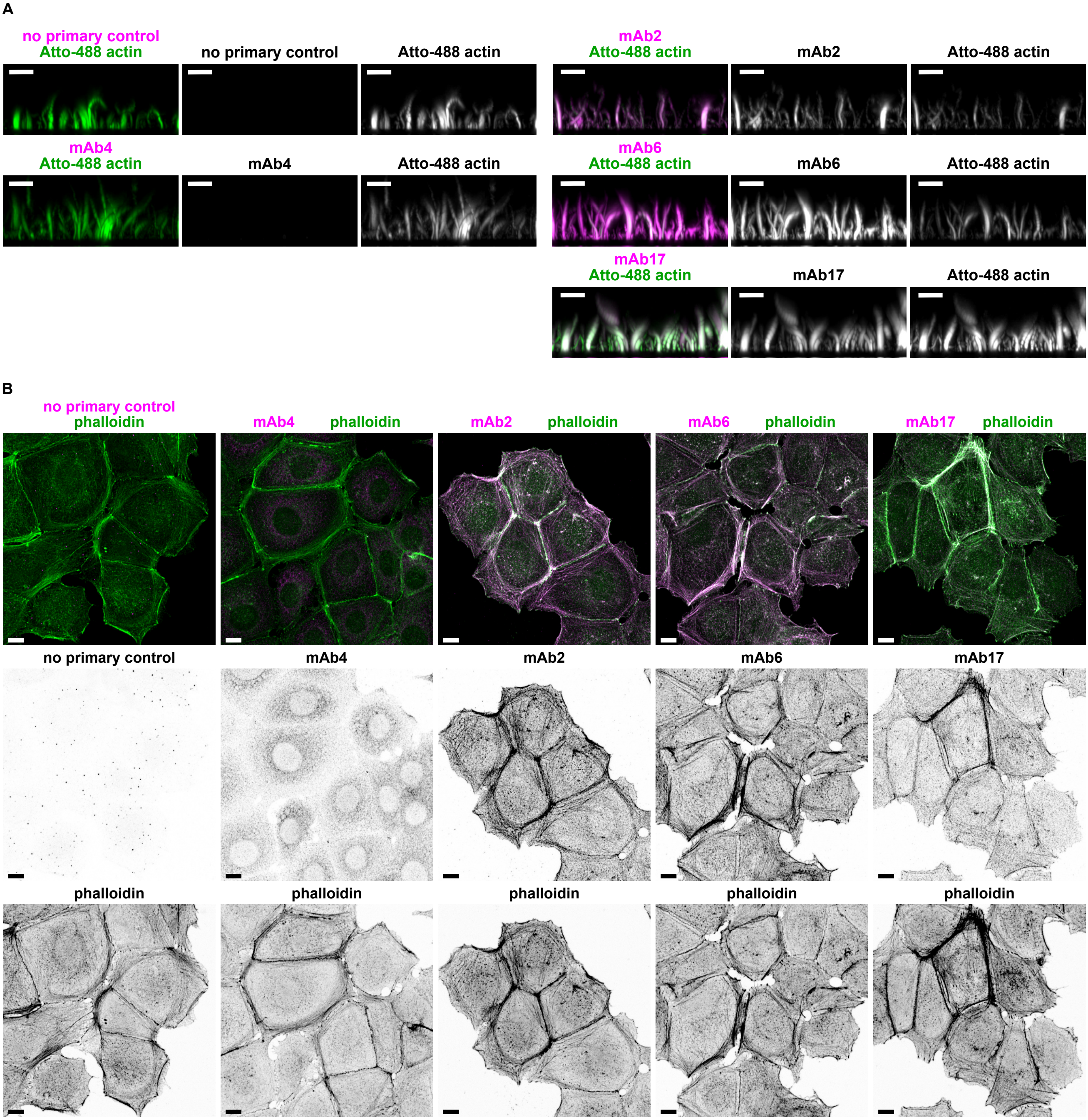
A
no primary control
Atto-488 actin
no primary control
Atto-488 actin
mAb2
Atto-488 actin
mAb2
Atto-488 actin
mAb4
Atto-488 actin
mAb4
Atto-488 actin
mAb6
Atto-488 actin
mAb6
Atto-488 actin
mAb17
Atto-488 actin
mAb17
Atto-488 actin
B
no primary control
phalloidin
mAb4
phalloidin
mAb2
phalloidin
mAb6
phalloidin
mAb17
phalloidin
no primary control
mAb4
mAb2
mAb6
mAb17
phalloidin
phalloidin
phalloidin
phalloidin
phalloidin

Figure S2

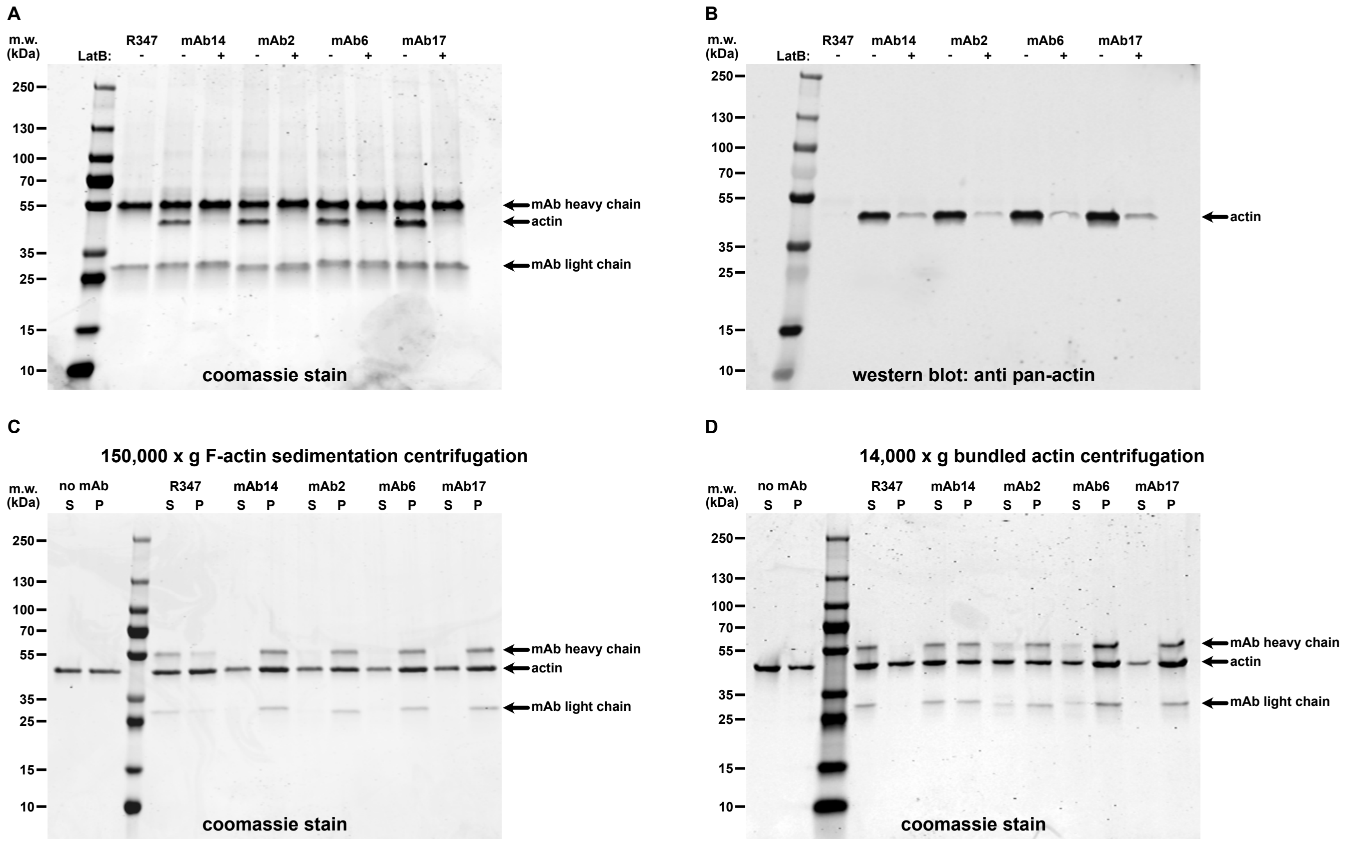

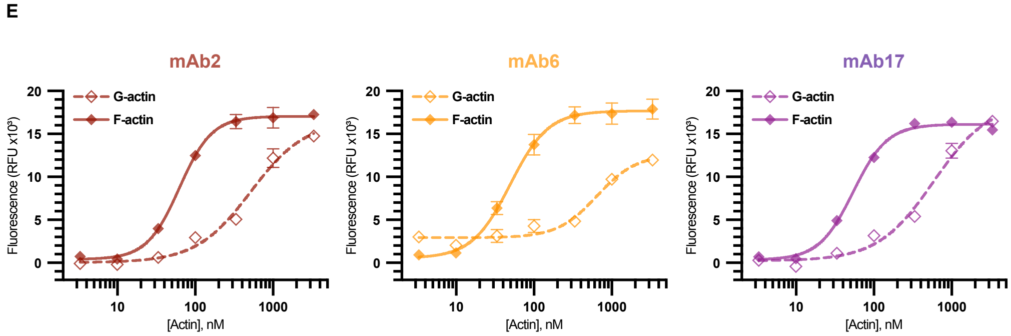

F

Antibody	F-actin EC50 (95% CI) / nM	G-actin EC50 (95% CI) / nM
mAb14	54.81 (47.96-62.63)	617.5 (344.4-12111)
mAb2	62.76 (52.29-75.09)	519.3 (359.8-1226)
mAb6	49.49 (36.92-65.75)	644.9 (444.3-3733)
mAb17	54.17 (46.41-63.61)	587.8 (432.6-1154)

Figure S3

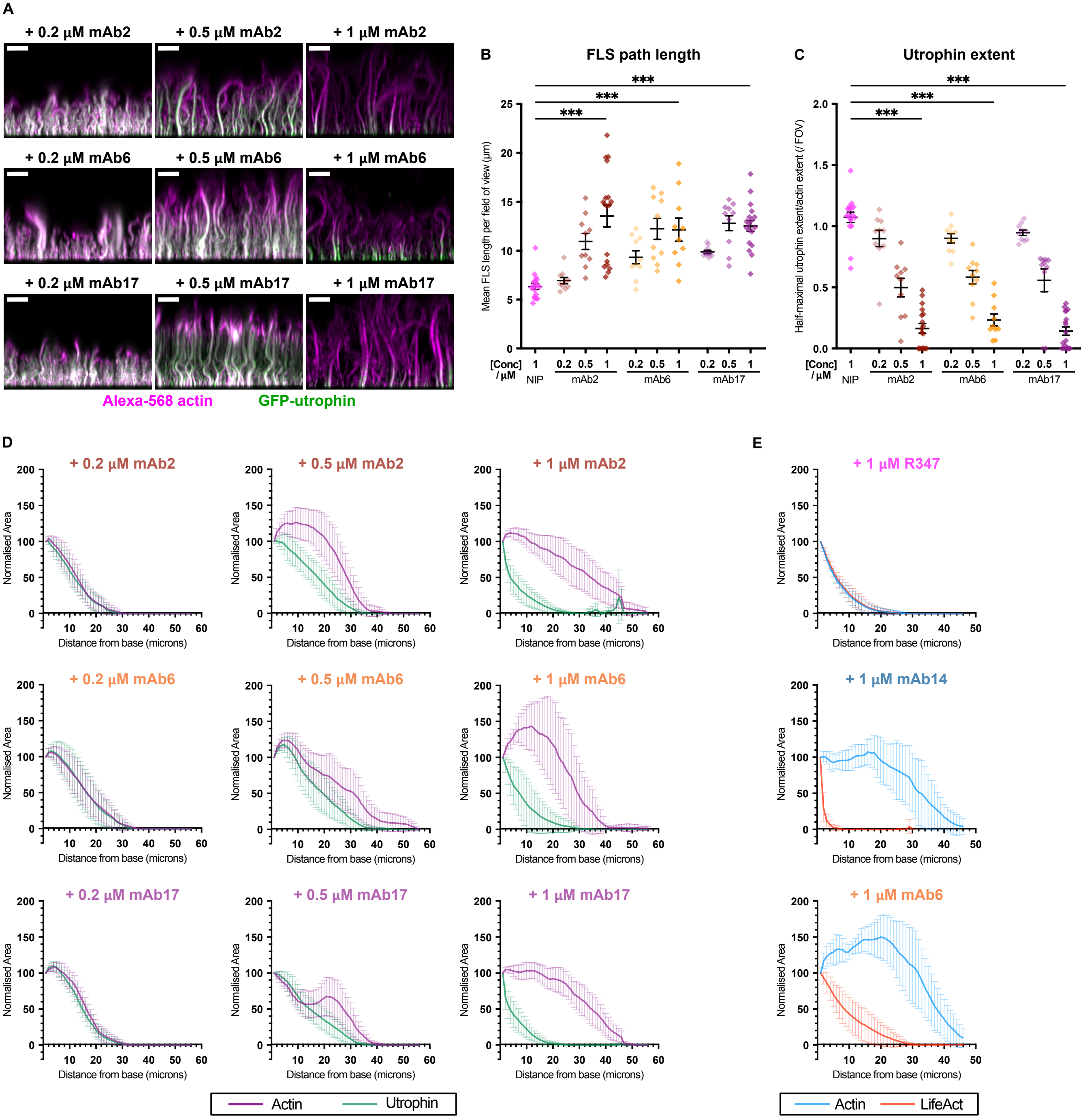
A
+ 0.2 µM mAb2
+ 0.5 µM mAb2
+ 1 µM mAb2
+ 0.2 µM mAb6
+ 0.5 µM mAb6
+ 1 µM mAb6
+ 0.2 µM mAb17
+ 0.5 µM mAb17
+ 1 µM mAb17
Alexa-568 actin
GFP-utrophin
B
FLS path length
Mean FLS length per field of view (µm)
***
[Conc] / µM
NIP
mAb2
mAb6
mAb17
C
Utrophin extent
Half-maximal utrophin extent/actin extent (/ FOV)
D
Normalised Area
Distance from base (microns)
Actin
Utrophin
E
+ 1 µM R347
+ 1 µM mAb14
+ 1 µM mAb6
Actin
LifeAct

Figure S4

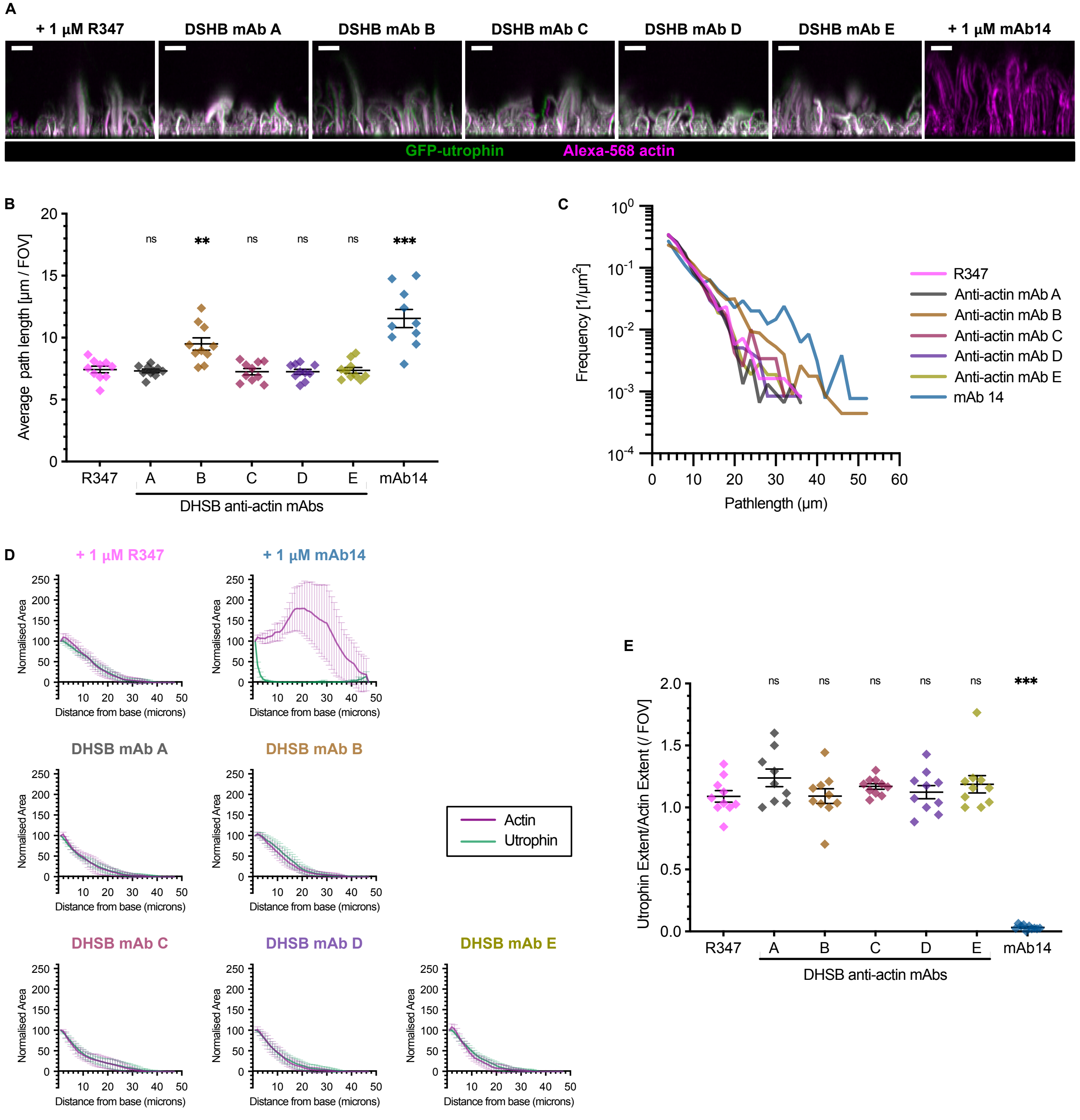

A
+ 1 µM R347
DSHB mAb A
DSHB mAb B
DSHB mAb C
DSHB mAb D
DSHB mAb E
+ 1 µM mAb14
GFP-utrophin
Alexa-568 actin
B
Average path length [µm / FOV]
ns
**
ns
ns
ns
***
R347
A
B
C
D
E
mAb14
DHSB anti-actin mAbs
C
Frequency [1/µm²]
Pathlength (µm)
R347
Anti-actin mAb A
Anti-actin mAb B
Anti-actin mAb C
Anti-actin mAb D
Anti-actin mAb E
mAb 14
D
+ 1 µM R347
+ 1 µM mAb14
DHSB mAb A
DHSB mAb B
DHSB mAb C
DHSB mAb D
DHSB mAb E
Normalised Area
Distance from base (microns)
Actin
Utrophin
E
Utrophin Extent/Actin Extent (/ FOV)
ns
ns
ns
ns
ns
***
R347
A
B
C
D
E
mAb14
DHSB anti-actin mAbs

Figure S5

A

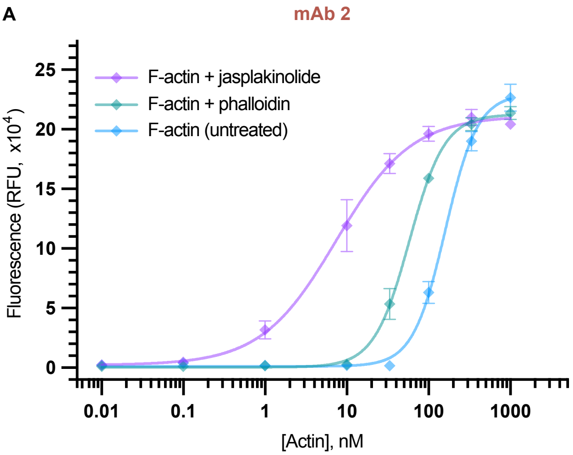

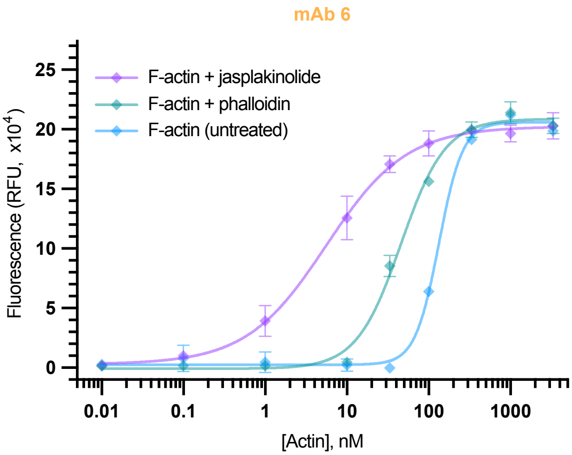

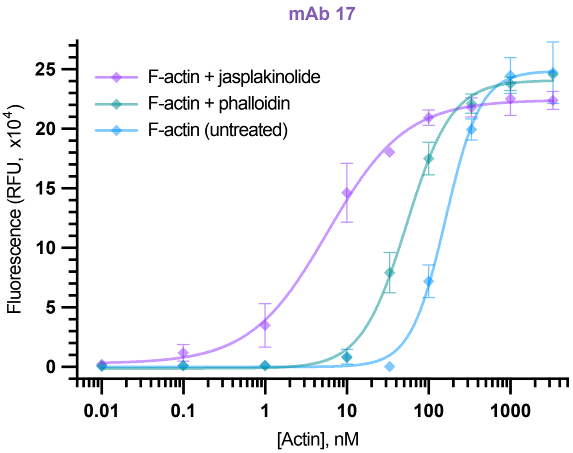

B

EC50 (95% CI) / nM				
**Antibody**	F-actin + jasplakinolide	F-actin + phalloidin	F-actin (untreated)	G-actin (untreated)
mAb14	6.187 (4.658-8.069)	50.87 (45.67-56.78)	166.8 (155.0-179.7)	n/a
mAb2	7.422 (5.744-9.411)	58.22 (54.22-62.53)	160.2 (145.5-176.7)	n/a
mAb6	5.587 (4.218-7.273)	45.84 (40.53-52.03)	133.3 (121.0-145.7)	n/a
mAb17	5.857 (4.286-7.848)	53.84 (47.89-60.70)	161.5 (140.2-186.4)	n/a
pan-actin mAb	36.22 (22.71-75.97)	68.83 (45.5-124.9)	26.49 (21.68-33.10)	26.49 (13.20-20.10)

Figure S6

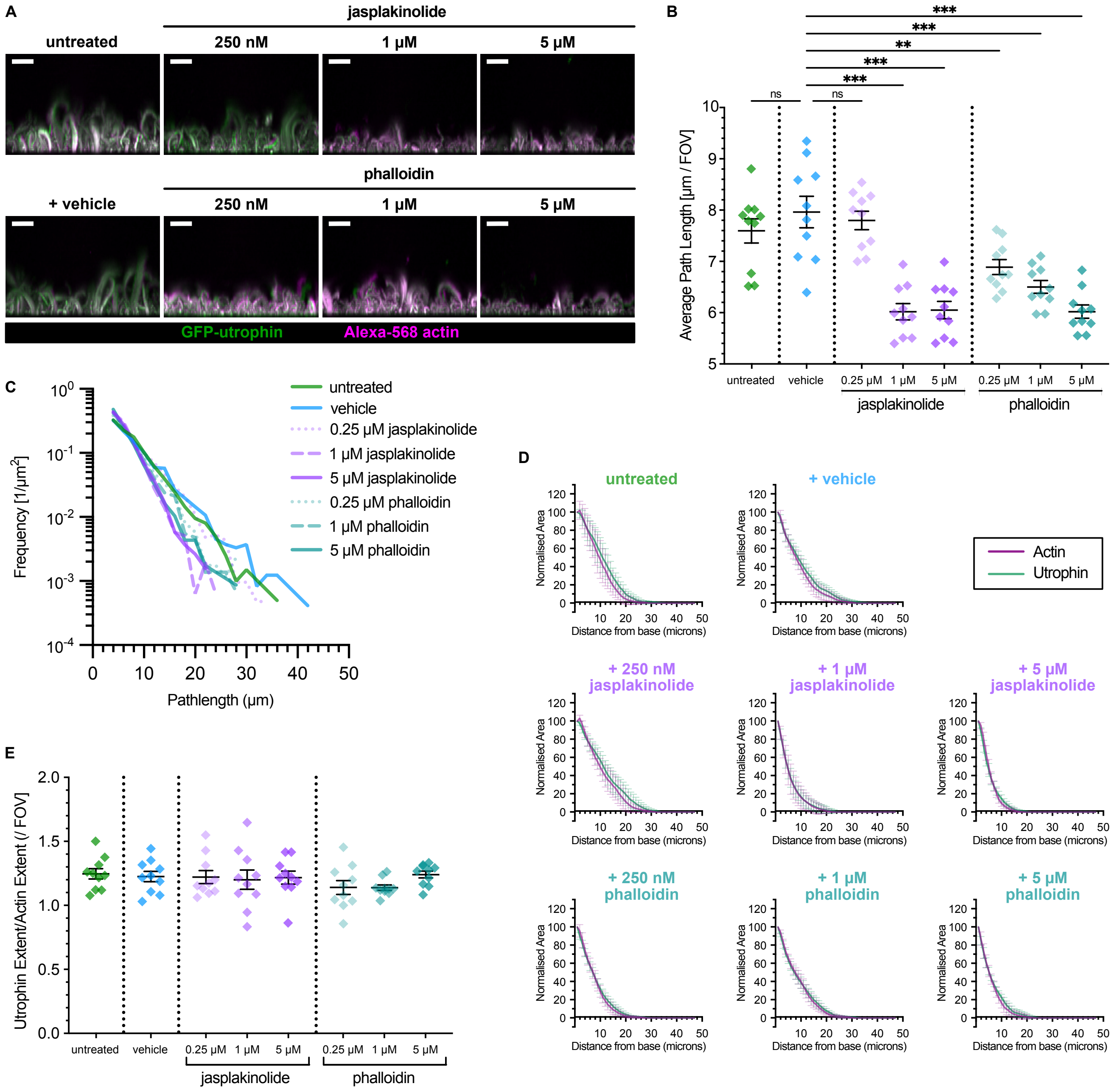

A
jasplakinolide
untreated
250 nM
1 μM
5 μM
phalloidin
+ vehicle
250 nM
1 μM
5 μM
GFP-utrophin
Alexa-568 actin
B
***
***
**
***
***
ns
ns
Average Path Length [μm / FOV]
untreated
vehicle
0.25 μM
1 μM
5 μM
0.25 μM
1 μM
5 μM
jasplakinolide
phalloidin
C
untreated
vehicle
0.25 μM jasplakinolide
1 μM jasplakinolide
5 μM jasplakinolide
0.25 μM phalloidin
1 μM phalloidin
5 μM phalloidin
Frequency [1/μm²]
Pathlength (μm)
D
untreated
+ vehicle
Actin
Utrophin
+ 250 nM jasplakinolide
+ 1 μM jasplakinolide
+ 5 μM jasplakinolide
+ 250 nM phalloidin
+ 1 μM phalloidin
+ 5 μM phalloidin
Normalised Area
Distance from base (microns)
E
Utrophin Extent/Actin Extent (/ FOV)
untreated
vehicle
0.25 μM
1 μM
5 μM
0.25 μM
1 μM
5 μM
jasplakinolide
phalloidin

Figure S7

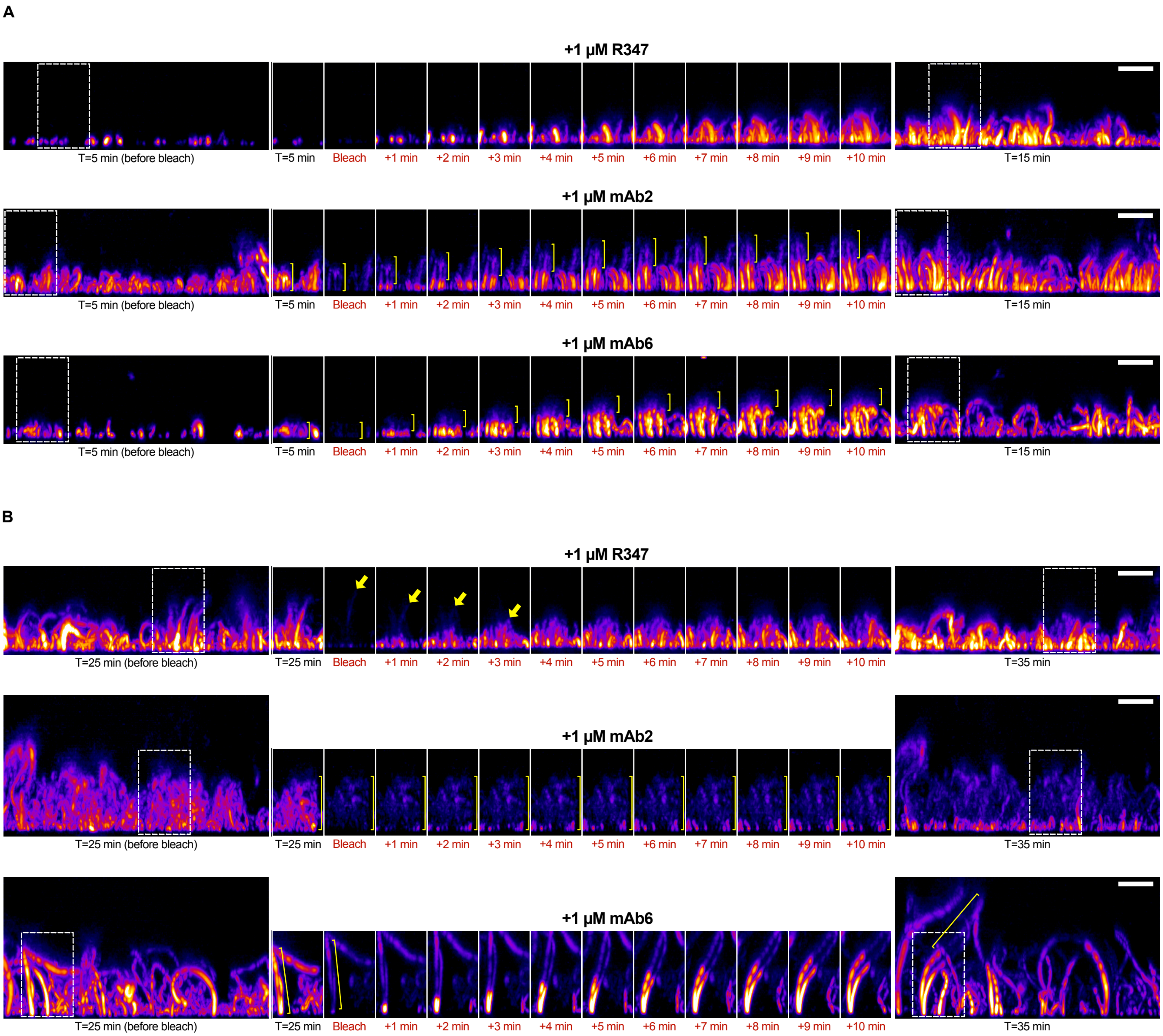
A
+1 μM R347
T=5 min (before bleach)
T=5 min Bleach +1 min +2 min +3 min +4 min +5 min +6 min +7 min +8 min +9 min +10 min
T=15 min
+1 μM mAb2
T=5 min (before bleach)
T=5 min Bleach +1 min +2 min +3 min +4 min +5 min +6 min +7 min +8 min +9 min +10 min
T=15 min
+1 μM mAb6
T=5 min (before bleach)
T=5 min Bleach +1 min +2 min +3 min +4 min +5 min +6 min +7 min +8 min +9 min +10 min
T=15 min
B
+1 μM R347
T=25 min (before bleach)
T=25 min Bleach +1 min +2 min +3 min +4 min +5 min +6 min +7 min +8 min +9 min +10 min
T=35 min
+1 μM mAb2
T=25 min (before bleach)
T=25 min Bleach +1 min +2 min +3 min +4 min +5 min +6 min +7 min +8 min +9 min +10 min
T=35 min
+1 μM mAb6
T=25 min (before bleach)
T=25 min Bleach +1 min +2 min +3 min +4 min +5 min +6 min +7 min +8 min +9 min +10 min
T=35 min

Figure S8

Table S1. Overview of data & statistical testing

<table>
<tr><th rowspan="2">Figure</th><th rowspan="2">Test</th><th colspan="3">Condition 1</th><th rowspan="2"></th><th colspan="3">Condition 2</th><th rowspan="2">P Value</th></tr>
<tr><th>Name</th><th>N (FOV)</th><th>Mean ± SEM [µm]</th><th>Name</th><th>N (FOV)</th><th>Mean ± SEM [µm]</th></tr>
<tr><td rowspan="5">Figure 1B

Mean FLS length per field of view (µm)</td><td rowspan="5">Ordinary one-way ANOVA w/ Sidak's multiple comparison test

Overall: P<0.001 [***]</td><td rowspan="5">R347</td><td rowspan="5">25</td><td rowspan="5">7.41 ± 0.131</td><td rowspan="5">Vs.</td><td>R347</td><td>20</td><td>6.54 ± 0.252</td><td>0.539 [ns]</td></tr>
<tr><td>mAb2</td><td>20</td><td>12.3 ± 0.505</td><td>< 0.001 [***]</td></tr>
<tr><td>mAb6</td><td>25</td><td>13.2 ± 0.597</td><td>< 0.001 [***]</td></tr>
<tr><td>mAb14</td><td>25</td><td>10.6 ± 0.289</td><td>< 0.001 [***]</td></tr>
<tr><td>mAb17</td><td>20</td><td>9.65 ± 0.506</td><td>0.001 [**]</td></tr>
</table>

<table>
<tr><th rowspan="2">Figure</th><th rowspan="2">Test</th><th colspan="3">Condition 1</th><th rowspan="2"></th><th colspan="3">Condition 2</th><th rowspan="2">P Value</th></tr>
<tr><th>Name</th><th>N (FOV)</th><th>Mean ± SEM [µm]</th><th>Name</th><th>N (FOV)</th><th>Mean ± SEM [µm]</th></tr>
<tr><td rowspan="11">Figure 3B

Mean FLS length per field of view (µm)</td><td rowspan="11">Ordinary one-way ANOVA w/ Sidak's multiple comparison test

Overall: P<0.001 [***]</td><td rowspan="3">0.2 µM NIP228</td><td rowspan="3">8</td><td rowspan="3">8.18 ± 0.445</td><td rowspan="11">Vs.</td><td>0.2 µM mAb14</td><td>8</td><td>11.4 ± 0.197</td><td>0.002 [**]</td></tr>
<tr><td>0.5 µM NIP228</td><td>8</td><td>7.65 ± 0.175</td><td>0.998 [ns]</td></tr>
<tr><td rowspan="2">1 µM NIP228</td><td rowspan="2">8</td><td rowspan="2">7.07 ± 0.211</td><td>0.996 [ns]</td></tr>
<tr><td rowspan="2">0.5 µM NIP228</td><td rowspan="2">8</td><td rowspan="2">7.65 ± 0.175</td><td>0.797 [ns]</td></tr>
<tr><td rowspan="2">0.5 µM mAb14</td><td rowspan="2">8</td><td rowspan="2">13.9 ± 0.991</td><td>< 0.001 [***]</td></tr>
<tr><td rowspan="2">0.2 µM mAb14</td><td rowspan="2">8</td><td rowspan="2">11.4 ± 0.197</td><td>0.018 [*]</td></tr>
<tr><td rowspan="2">1 µM mAb14</td><td rowspan="2">8</td><td rowspan="2">13.7 ± 0.723</td><td>0.046 [*]</td></tr>
<tr><td>0.5 µM mAb14</td><td>8</td><td>13.9 ± 0.991</td><td>> 0.999 [ns]</td></tr>
<tr><td rowspan="3">1 µM NIP228</td><td rowspan="3">8</td><td rowspan="3">7.07 ± 0.211</td><td>0.2 µM mAb14</td><td>8</td><td>11.4 ± 0.197</td><td>< 0.001 [***]</td></tr>
<tr><td>0.5 µM mAb14</td><td>8</td><td>13.9 ± 0.991</td><td>< 0.001 [***]</td></tr>
<tr><td>1 µM mAb14</td><td>8</td><td>13.7 ± 0.723</td><td>< 0.001 [***]</td></tr>
</table>

<table>
<tr><th rowspan="2">Figure</th><th rowspan="2">Test</th><th colspan="3">Condition 1</th><th rowspan="2"></th><th colspan="3">Condition 2</th><th rowspan="2">P Value</th></tr>
<tr><th>Name</th><th>N (FOV)</th><th>Mean ± SEM [AU]</th><th>Name</th><th>N (FOV)</th><th>Mean ± SEM [AU]</th></tr>
<tr><td rowspan="11">Figure 3D

Half-maximal utrophin extent / actin extent (/FOV)</td><td rowspan="11">Ordinary one-way ANOVA w/ Sidak's multiple comparison test

Overall: P<0.001 [***]</td><td rowspan="3">0.2 µM NIP228</td><td rowspan="3">8</td><td rowspan="3">1.11 ± 0.016</td><td></td><td>0.2 µM mAb14</td><td>8</td><td>0.99 ± 0.030</td><td>0.456 [ns]</td></tr>
<tr><td rowspan="10">Vs.</td><td>0.5 µM NIP228</td><td>8</td><td>1.13 ± 0.017</td><td>> 0.999 [ns]</td></tr>
<tr><td rowspan="2">1 µM NIP228</td><td rowspan="2">8</td><td rowspan="2">1.16 ± 0.067</td><td>> 0.997 [ns]</td></tr>
<tr><td rowspan="2">0.5 µM NIP228</td><td rowspan="2">8</td><td rowspan="2">1.13 ± 0.017</td><td>> 0.999 [ns]</td></tr>
<tr><td rowspan="2">0.5 µM mAb14</td><td rowspan="2">8</td><td rowspan="2">0.73 ± 0.056</td><td>< 0.001 [***]</td></tr>
<tr><td rowspan="2">0.2 µM mAb14</td><td rowspan="2">8</td><td rowspan="2">0.99 ± 0.030</td><td>0.002 [**]</td></tr>
<tr><td rowspan="2">1 µM mAb14</td><td rowspan="2">8</td><td rowspan="2">0.25 ± 0.053</td><td>< 0.001 [***]</td></tr>
<tr><td>0.5 µM mAb14</td><td>8</td><td>0.73 ± 0.056</td><td>< 0.001 [***]</td></tr>
<tr><td rowspan="3">1 µM NIP228</td><td rowspan="3">8</td><td rowspan="3">1.16 ± 0.067</td><td>0.2 µM mAb14</td><td>8</td><td>0.99 ± 0.030</td><td>0.082 [ns]</td></tr>
<tr><td>0.5 µM mAb14</td><td>8</td><td>0.73 ± 0.056</td><td>< 0.001 [***]</td></tr>
<tr><td>1 µM mAb14</td><td>8</td><td>0.25 ± 0.053</td><td>< 0.001 [***]</td></tr>
</table>

Figure	Test	Condition 1				Condition 2			P Value
		Name	N (FOV)	Mean ± SEM [AU]		Name	N (FOV)	Mean ± SEM [AU]	
**Figure 3F**<br>Half-maximal LifeAct extent / actin extent (/FOV)	Ordinary one-way ANOVA w/ Sidak's multiple comparison test<br>Overall: P<0.001 [***]	R347	8	1.05 ± 0.027	Vs.	mAb14	6	0.00 ± 0.00	< 0.001 [***]
						mAb6	8	0.14 ± 0.046	< 0.001 [***]

Figure	Test	Condition 1				Condition 2			P Value
		Name	N (FOV)	Mean ± SEM [AU]		Name	N (FOV)	Mean ± SEM [AU]	
**Figure 6E**<br>Half-maximal utrophin extent / actin extent (/FOV)	Ordinary one-way ANOVA w/ Sidak's multiple comparison test<br>Overall: P<0.001 [***]	**R347 mAb only**	10	1.07 ± 0.024	Vs.	**mAb14 mAb only**	10	0.18 ± 0.028	< 0.001 [***]
						**R347 + actin**	9	1.09 ± 0.011	> 0.999 [ns]
						**R347 + cofilin**	10	1.16 ± 0.063	0.592 [ns]
						**R347 + actin & cofilin**	9	1.13 ± 0.001	0.974 [ns]
		**R347 + actin**	9	1.09 ± 0.011					> 0.999 [ns]
		**R347 + cofilin**	10	1.16 ± 0.063					> 0.999 [ns]
		**mAb14 mAb only**	10	0.18 ± 0.028		**mAb14 + actin**	10	0.48 ± 0.02	< 0.001 [***]
						**mAb14 + cofilin**	10	0.022 ± 0.008	0.058 [ns]
						**mAb14 + actin & cofilin**	10	0.53 ± 0.070	< 0.001 [***]
		**mAb14 + actin**	10	0.48 ± 0.02					0.995
		**mAb14 + cofilin**	10	0.022 ± 0.008					< 0.001 [***]

Figure	Test	Condition 1				Condition 2			P Value
		Name	N (FOV)	Mean ± SEM [µm]		Name	N (FOV)	Mean ± SEM [µm]	
**Extended Data Figure 4B**<br><br>Mean FLS length per field of view (µm)	Ordinary one-way ANOVA w/ Sidak's multiple comparison test<br><br>Overall: P<0.001 [***]	1 µM NIP228	18	6.36 ± 0.303	Vs.	0.2 µM mAb2	10	6.95 ± 0.320	> 0.999 [ns]
						0.5 µM mAb2	10	11.0 ± 0.824	0.001 [**]
						1 µM mAb2	18	13.5 ± 1.11	< 0.001 [***]
						0.2 µM mAb6	10	9.34 ± 0.665	0.133 [ns]
						0.5 µM mAb6	10	12.2 ± 1.07	< 0.001 [***]
						1 µM mAb6	10	12.2 ± 1.17	< 0.001 [***]
						0.2 µM mAb17	10	9.89 ± 0.170	0.032 [*]
						0.5 µM mAb17	10	12.8 ± 0.763	< 0.001 [***]
						1 µM mAb17	18	12.6 ± 0.562	< 0.001 [***]
		0.2 µM mAb2	10	6.95 ± 0.320		0.5 µM mAb2	11	10.7 ± 0.776	0.031 [*]
						1 µM mAb2	18	13.8 ± 1.14	< 0.001 [***]
		0.5 µM mAb2	10	10.7 ± 0.776					0.317 [ns]
		0.2 µM mAb6	10	9.34 ± 0.665		0.5 µM mAb6	10	12.2 ± 1.07	0.334 [ns]
						1 µM mAb6	10	12.2 ± 1.17	0.384 [ns]
		0.5 µM mAb6	10	12.2 ± 1.07					> 0.999 [ns]
		0.2 µM mAb17	10	9.89 ± 0.170		0.5 µM mAb17	10	12.8 ± 0.763	0.316 [ns]
						1 µM mAb17	18	12.6 ± 0.562	0.272 [ns]
		0.5 µM mAb17	10	12.8 ± 0.763					> 0.999 [ns]

Figure	Test	Condition 1				Condition 2			P Value
		Name	N (FOV)	Mean ± SEM [AU]		Name	N (FOV)	Mean ± SEM [AU]	
**Extended Data Figure 4C**<br><br>Half-maximal utrophin Extent / Actin Extent (/FOV)	Ordinary one-way ANOVA w/ Sidak's multiple comparison test<br><br>Overall: P<0.001 [***]	1 µM NIP228	17	1.07 ± 0. 042	Vs.	0.2 µM mAb2	10	0.90 ± 0. 068	0.274 [ns]
						0.5 µM mAb2	10	0.50 ± 0. 076	< 0.001 [***]
						1 µM mAb2	18	0.17 ± 0. 039	< 0.001 [***]
						0.2 µM mAb6	10	0.90 ± 0. 037	0.284 [ns]
						0.5 µM mAb6	10	0.58 ± 0. 055	< 0.001 [***]
						1 µM mAb6	10	0.23 ± 0. 049	< 0.001 [***]
						0.2 µM mAb17	10	0.95 ± 0. 022	0.785 [ns]
						0.5 µM mAb17	10	0.56 ± 0. 094	< 0.001 [***]
						1 µM mAb17	18	0.14 ± 0. 037	< 0.001 [***]
		0.2 µM mAb2	10	0.90 ± 0. 068		0.5 µM mAb2	10	0.50 ± 0. 076	< 0.001 [***]
						1 µM mAb2	18	0.17 ± 0. 039	< 0.001 [***]
		0.5 µM mAb2	10	0.50 ± 0. 076					< 0.001 [***]
		0.2 µM mAb6	10	0.90 ± 0. 037		0.5 µM mAb6	10	0.58 ± 0. 055	0.003 [**]
						1 µM mAb6	10	0.23 ± 0. 049	< 0.001 [***]
		0.5 µM mAb6	10	0.58 ± 0. 055					< 0.001 [***]
		0.2 µM mAb17	10	0.95 ± 0. 022		0.5 µM mAb17	10	0.56 ± 0. 094	< 0.001 [***]
						1 µM mAb17	18	0.14 ± 0. 037	< 0.001 [***]
		0.5 µM mAb17	10	0.56 ± 0. 094					< 0.001 [***]

Figure	Test	Condition 1			Vs.	Condition 2			P Value
		Name	N (FOV)	Mean ± SEM [µm]		Name	N (FOV)	Mean ± SEM [µm]	
**Extended Data Figure 5B**<br><br>Mean FLS length per field of view (µm)	Ordinary one-way ANOVA w/ Sidak's multiple comparison test<br><br>Overall: P<0.001 [***]	R347	10	7.43 ± 0.262	Vs.	DHSB mAb A	10	7.32 ± 0.140	> 0.999 [ns]
						DHSB mAb B	10	9.49 ± 0.490	0.002 [**]
						DHSB mAb C	10	7.26 ± 0.247	> 0.999 [ns]
						DHSB mAb D	10	7.25 ± 0.203	> 0.999 [ns]
						DHSB mAb E	10	7.36 ± 0.239	> 0.999 [ns]
						mAb14	10	11.6 ± 0.736	< 0.001 [***]

Figure	Test	Condition 1			Vs.	Condition 2			P Value
		Name	N (FOV)	Mean ± SEM [AU]		Name	N (FOV)	Mean ± SEM [AU]	
**Extended Data Figure 5E**<br><br>Half-maximal utrophin extent / actin extent (/FOV)	Ordinary one-way ANOVA w/ Sidak's multiple comparison test<br><br>Overall: P<0.001 [***]	R347	10	1.09 ± 0.046	Vs.	DHSB mAb A	10	1.24 ± 0.072	0.258 [ns]
						DHSB mAb B	9	1.09 ± 0.059	> 0.999 [ns]
						DHSB mAb C	10	1.17 ± 0. 022	0.853 [ns]
						DHSB mAb D	10	1.12 ± 0.053	0.998 [ns]
						DHSB mAb E	10	1.19 ± 0.070	0.695 [ns]
						mAb14	10	0.033 ± 0.006	< 0.001 [***]

Figure	Test	Condition 1			Vs.	Condition 2			P Value
		Name	N (FOV)	Mean ± SEM [µm]		Name	N (FOV)	Mean ± SEM [µm]	
**Extended Data Figure 7B**<br><br>Mean FLS length per field of view (µm)	Ordinary one-way ANOVA w/ Sidak's multiple comparison test<br><br>Overall: P<0.001 [***]	Vehicle	10	7.96 ± 0.307	Vs.	Untreated	10	7.60 ± 0.237	0.743 [ns]
						+ 250 nM JSP	10	7.80 ± 0.180	0.996 [ns]
						+ 1 µM JSP	10	6.02 ± 0.158	< 0.001 [***]
						+ 5 µM JSP	10	6.05 ± 0.167	< 0.001 [***]
						+ 250 nM PHL	10	6.89 ± 0.147	> 0.999 [ns]
						+ 1 µM PHL	10	6.50 ± 0.126	0.001 [**]
						+ 5 µM PHL	10	6.02 ± 0.130	< 0.001 [***]
		JSP = Jasplakinolide				PHL = Phalloidin			

Figure	Test	Condition 1			Vs.	Condition 2			P Value
		Name	N (FOV)	Mean ± SEM [AU]		Name	N (FOV)	Mean ± SEM [AU]	
**Extended Data Figure 7E**<br><br>Half-maximal utrophin extent / actin extent (/FOV)	Ordinary one-way ANOVA w/ Sidak's multiple comparison test<br><br>Overall: P=0.598 [ns]	Vehicle	10	1.23 ± 0.041	Vs.	Untreated	10	1.25 ± 0.041	> 0.999 [ns]
						+ 250 nM JSP	10	1.22 ± 0.050	> 0.999 [ns]
						+ 1 µM JSP	10	1.20 ± 0. 075	> 0.999 [ns]
						+ 5 µM JSP	10	1.22 ± 0.050	> 0.999 [ns]
						+ 250 nM PHL	10	1.14 ± 0.055	0.805 [ns]
						+ 1 µM PHL	10	1.14 ± 0.021	0.799 [ns]
						+ 5 µM PHL	10	1.24 ± 0.026	> 0.999 [ns]
		JSP = Jasplakinolide				PHL = Phalloidin			
